# Relationship between peripheral blood *miR-146b-5p* levels and lymphocyte subsets in patients with sepsis and its predictive value for prognosis: a single-center study

**DOI:** 10.3389/fimmu.2025.1608576

**Published:** 2025-06-26

**Authors:** Sha Cheng, Jiaojiao Wang, Pengfei Wang, Xianli Long, Huiling Liu, Xueyin Li, Xiaowei Zhang, Xuemei Zhao, Hang Sun, Chuanxin Wu

**Affiliations:** ^1^ Key Laboratory of Molecular Biology for Infectious Diseases, Ministry of Education, Institute for Viral Hepatitis, The Second Affiliated Hospital of Chongqing Medical University, Chongqing, China; ^2^ Department of Respiratory and Critical Care Medicine, Nanfang Hospital, Southern Medical University, Guangzhou, China; ^3^ Department of Intensive Care Unit, The Second Affiliated Hospital, Chongqing Medical University, Chongqing, China; ^4^ Department of Hepatobiliary Surgery, The Second Affiliated Hospital, Chongqing Medical University, Chongqing, China

**Keywords:** diagnosis and prognosis of sepsis, immunomodulation, lymphocyte subsets, *miR-146b-5p*, sepsis

## Abstract

**Background:**

Sepsis is a life-threatening disease with challenges in clinical management due to delayed diagnosis and immunosuppression. Lymphopenia is a key prognostic indicator in sepsis. microRNAs (miRNAs) are recognized as key immune modulators affecting all stages of inflammation. In this study, we investigated the expression of *miR-146b-5p* and lymphocyte subsets in sepsis patients and analyzed their correlation with the aim of establishing their combined predictive value for clinical outcomes and advancing personalized treatment strategies.

**Methods:**

From January 2020 to July 2024, we enrolled 191 patients diagnosed with sepsis at the ICU of the Second Affiliated Hospital of Chongqing Medical University and collected basic clinical data. These patients were categorized into two groups: nonsurvivors (n = 117) and survivors (n = 74). Correlation analysis was employed to analyze the correlation between *miR-146b-5p* and lymphocyte subsets with disease severity. Binary logistic regression and Cox regression analyses were employed to identify independent risk factors influencing the prognosis of sepsis. The predictive value of *miR-146b-5p* and lymphocyte subsets for sepsis prognosis was assessed using receiver operating characteristic (ROC) curves.

**Results:**

*miR-146b-5p* expression was significantly lower in sepsis patients compared to HD group, with levels in the nonsurvivor group being lower than those in the survivor group. Survival curves for *miR-146b-5p* indicated that lower levels of *miR-146b-5p* (<0.272) were associated with a higher mortality rate (HR 3.063). The absolute counts of lymphocytes (Lym), CD3^+^ T cells, CD4^+^ T cells, and CD8^+^ T cells were significantly lower in the sepsis group compared to the HD group. Testing lymphocyte counts at different time points revealed that absolute counts of CD3^+^ T cells, CD4^+^ T cells, and CD8^+^ T cells were consistently lower in the sepsis group across all time intervals. *miR-146b-5p* levels were predictive of patient prognosis, with the combination of *miR-146b-5p* and APACHE II scores yielding the highest AUC.

**Conclusion:**

The early-stage sepsis-associated downregulation of *miR-146b-5p* serves as a promising biomarker for severity stratification and prognostic evaluation. The combination of *miR-146b-5p* with APACHE II scores enhances diagnostic accuracy. Additionally, dynamic monitoring of lymphocyte subsets may facilitate the evaluation of immune status and guide personalized treatment strategies.

## 1 Introduction

Sepsis is a life-threatening organ dysfunction caused by the dysregulated host immune response to infection and is an important global health problem (1). In 2017, an estimated 48.9 million incident cases of sepsis and 11 million sepsis-related deaths (representing 19.7% of all global deaths) were reported worldwide (2). Despite advances in clinical management of sepsis, sepsis remains the leading cause of death in the intensive care unit (ICU), with long-term mortality rates ranging from 40% to 80% (3–5). Moreover, sepsis treatment imposes a heavy burden on healthcare resources and significantly affects patients’ quality of life. Early diagnosis of sepsis is challenging and immune dysregulation during the course of the disease often leads to immunosuppression, which increases the risk of secondary infections and death (6–10). Therefore, early and accurate prediction of the patient’s condition and prognosis is of paramount clinical importance (11, 12).

The immune system in sepsis patients is severely compromised, marked by a loss of innate immune function and subsequent suppression of adaptive immunity (13, 14). Lymphopenia, characterized by a reduction in lymphocyte counts, is commonly observed in sepsis, with a significant depletion of CD4^+^ T cells (15). This depletion is strongly associated with increased mortality, underscoring the crucial role of CD4^+^ T cells as predictive biomarkers and potential therapeutic targets (16).

With the in-depth study of immune mechanisms in sepsis, microRNAs (miRNAs) have emerged as key regulators of immune responses. MiRNAs are a class of small, noncoding RNAs that usually negatively regulate gene expression by inducing messenger RNA (mRNA) cleavage or repressing translation (17). Aberrant miRNA expression has been observed in sepsis, indicating their potential utility in diagnosis and prognosis (18). In recent years, many studies have shown that miRNAs can be used as sepsis biomarkers, offering significant value in disease diagnosis, severity assessment and prognostic evaluation (19–24). MiRNAs are recognized as crucial immune regulators that modulate various phases of inflammation (23, 25), and their dysregulation has been reported in various autoimmune and autoinflammatory diseases, including rheumatoid arthritis, systemic lupus erythematosus (SLE) and inflammatory bowel disease (IBD) (26–28). Using miRNA gene chip technology, we identified *miR-146b-5p* as a key regulator of inflammation, capable of downregulating pro-inflammatory factors such as TNF-α, IL-6, and HMGB1 in primary peritoneal macrophages of mice induced by *Candida albicans* (29). However, the role of *miR-146b-5p* in human sepsis remains unexplored.

This study aimed to investigate the expression and changes of *miR-146b-5p* and lymphocyte subsets in patients with sepsis. We sought to analyze the correlation between *miR-146b-5p* and lymphocyte subsets and to assess the predictive value for sepsis prognosis. By elucidating the roles of *miR-146b-5p* and lymphocyte subsets in sepsis, we aim to identify novel biomarkers for early diagnosis and prognostic assessment, ultimately facilitating the development of individualized therapeutic strategies for sepsis.

## 2 Materials and methods

### 2.1 Patients and healthy donors

This prospective cohort study included patients and healthy controls from the ICU of the Second Affiliated Hospital of Chongqing Medical University, China. The study was conducted in accordance with the Declaration of Helsinki and approved by the Human Research Ethics Committee of Chongqing Medical University (Scientific Ethics Pre-Review No. (2018) 110). All procedures adhered to the relevant ethical guidelines and regulations. We rigorously enforced a three-level consent mechanism. First, informed consent was obtained from participants if they were able to provide written informed consent. Second, we rigorously sought informed consent from guardians (e.g., spouses, children) before conducting the study in patients who were unconscious or unconscious. Third, if the patients had no guardians, we would not take such patients as research subjects, and our study strictly complied with the requirements of clinical research medical ethics. A total of 191 consecutive patients aged ≥18 years old were admitted to the ICU between January 2020 and July 2024. Sepsis was diagnosed according to the Sepsis-3 criteria, which defines sepsis as life-threatening organ dysfunction caused by a dysregulated host response to infection. Organ dysfunction was determined by an acute increase of 2 or more points in the Sequential Organ Failure Assessment (SOFA) score. The exclusion criteria included: malignant tumor, autoimmune disease, hematopoietic system disease and immunodeficiency disease; use of radiotherapy, chemotherapy and immunosuppressant within half a year. Healthy donors (HD) were randomly selected from outpatients at the Second Affiliated Hospital of Chongqing Medical University to serve as controls. In order to ensure that the healthy control group and the case group were matched on key characteristics, we took the following approach: (1) Age matching: Individuals with a similar age distribution to the case group were screened to reduce the potential effect of age differences. (2) Gender matching: the gender composition of the healthy controls was determined according to the sex ratio of the case group to ensure that the sex ratio was consistent. (3) Basic health status matching: healthy donors were strictly screened and those with a history of chronic diseases were excluded to ensure the comparability of health status with the case group. Informed consent was obtained from all participants providing samples. All sepsis patients were treated in accordance with the guidelines of the Surviving Sepsis Campaign.

**Figure 1:**
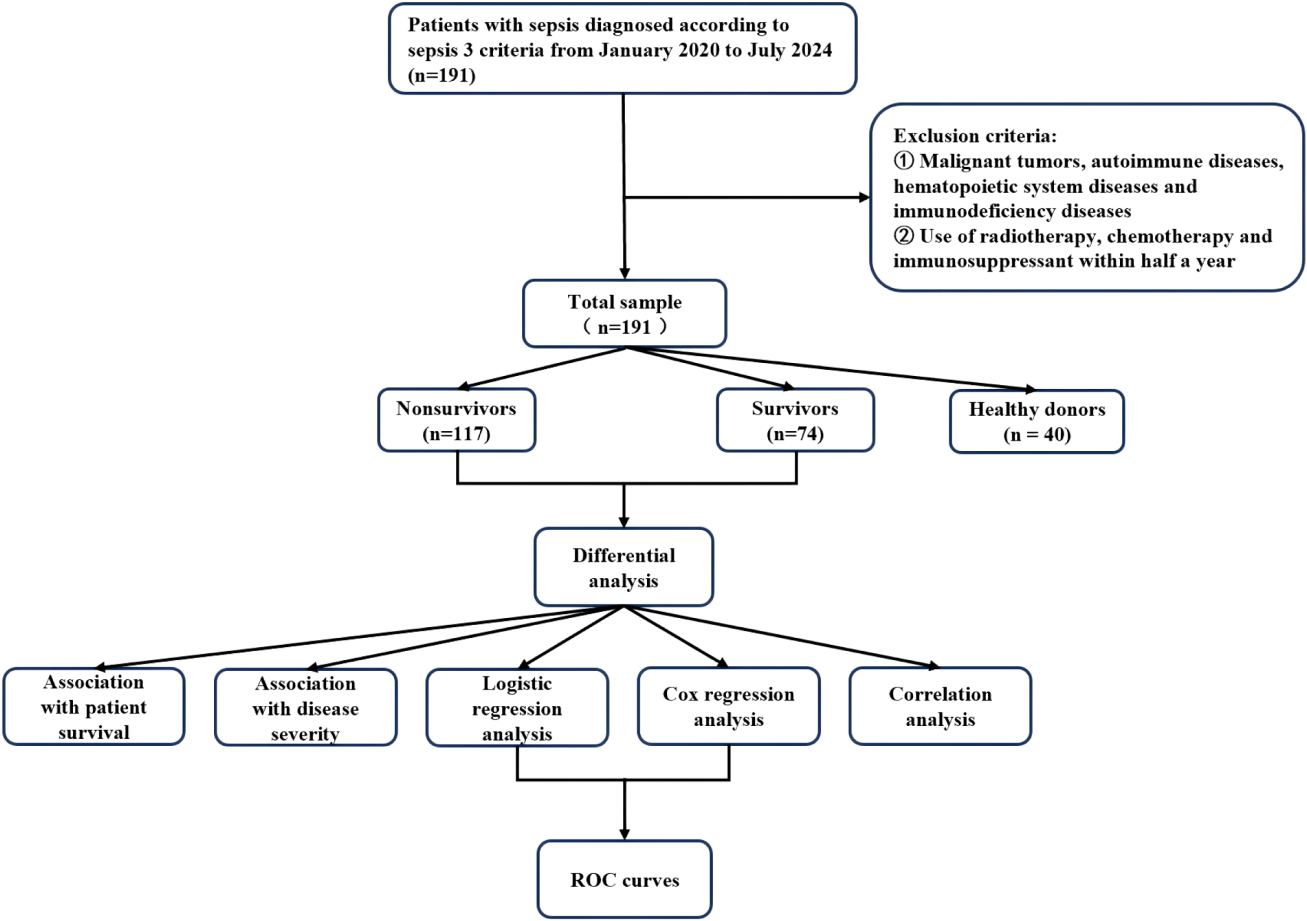
Flowchart of patient selection, grouping and statistical analysis in this study.

For the two indicators of main interest in this study, miR146b-5p and lymphocyte subsets, there was no missing data for miR146b-5p, and 12 (6%) were missing data for lymphocyte subsets. For the baseline indicators, 76 (39%) were missing data for IL-1β, 114 (59%) for IL-2, 70 (36%) for IL-6, 146 (76%) for IL-8, and 70 (36%) for IL-10. There were 71 (37%) missing data for TNF-α, 15 (7%) missing data for CRP, 21 (10%) missing data for PCT, 97 (50%) missing data for SOFA score, and 46 (18%) missing data for APACHE II score. There were 16 cases (8%) missing data for white blood cells, 15 cases (7%) missing data for neutrophils, 15 cases (7%) missing data for lymphocytes, and 15 cases (7%) missing data for monocytes. For continuous variables with a proportion of missing data (5% - 10%) that were missing at random, we used mean imputation to preserve the completeness of the data. Multiple imputation was used for variables with a high proportion of missing data (>10%) and a complex missing mechanism. The patient selection flow chart, grouping, and data analysis process are presented in **Figure 1**.

### 2.2 Sample collection

Blood samples were collected at four time points during patient admission: days 1-3 (D1-3), days 4-7 (D4-7), days 8-12 (D8-12), and days 13-21 (D13-21). These samples were used for the analysis of miR-146b-5p expression and lymphocyte subsets. At admission, the following baseline demographic and clinical information was recorded: age, gender, Acute Physiology and Chronic Health Evaluation (APACHE II) scores, SOFA scores, complete blood count results, and cytokine levels. The primary endpoint was 28-day mortality.

Additional clinical data were collected throughout the study, including vital signs, medical history, laboratory test results, diagnoses, and clinical outcomes. The SOFA and APACHE II scores were calculated within the first 24 hours following the diagnosis of sepsis. In addition, 2 mL of venous blood was collected in EDTA vacutainers within 24 hours of sepsis diagnosis. Peripheral blood mononuclear cells (PBMCs) were isolated from these blood samples using density gradient centrifugation with Ficoll-Hypaque (TBD Science; Tianjin, China), following the manufacturer’s instructions.

### 2.3 Quantitative real-time polymerase chain reaction analysis

Total RNA was isolated from cells using TRIzol Reagent (Thermo Fisher Scientific). One milliliter of TRIzol and 200 microliters of chloroform were added to the sample and mixed vigorously for 15 seconds, followed by incubation on ice for 10 minutes. The samples were centrifuged at 4°C for 10 minutes at 12,000 rpm to achieve complete phase separation. The upper aqueous phase was collected, and isopropanol was added in a 1:1 ratio to a new centrifuge tube, which was mixed gently and incubated on ice for an additional 10 minutes. After centrifugation at 4°C for 10 minutes at 12,000 rpm, the resulting pellets were washed once with 75% ethanol. The precipitated RNA was resuspended in 30 microliters of RNase-free water. To assess the quantity and quality of RNA, the samples were measured using a NanoDrop spectrophotometer (NanoDrop, Thermo) and analyzed by agarose gel electrophoresis. A total of 1 microgram of RNA was reverse transcribed into cDNA using the PrimeScript RT reagent kit (Takara, Dalian, China). Quantitative real-time PCR analysis was conducted using the SYBR Premix Ex Taq™ II kit (Takara, Dalian, China). Primers were purchased from Sangon, Shanghai, China. The primers for human *miR-146b-5p* were: 5’- CGCGTGAGAACTGAATTCCAT -3’(sense) and 5’-AGTGCAGGGTCCGAGGTATT -3’ (antisense), and U6 primers were 5’-AGAGAAGATTAGCATGGCCCCTG-3’ (sense) and 5’-AGTGCAGGGTCCGAGGTATT-3’ (antisense). The thermal cycling conditions for qPCR were as follows: 95°C for 30 s (step 1), 40 cycles of 95°C for 5 s, 60°C for 30 s (step 2); 95°C for 15 s, 65°C for 30 s, 95°C for 15 s (step 3), and then maintained at 4°C. The reactions were performed on a CFX Connect Sequence Detection System (Bio-Rad). The mRNA expression levels of target genes were normalized to U6 using the 2^-ΔΔCt^method where ΔCt = target gene Ct-U6 Ct and ΔΔCt = ΔCt treatment-ΔCt control. Three independent experiments were conducted in triplicate.

### 2.4 Determination of lymphocyte subsets

2 mL of whole blood was collected in ethylenediaminetetraacetic acid (EDTA)-containing tubes and analyzed within 6 hours of collection. PBMCs were stained with fluorochrome-conjugated antibodies specific for CD3, CD4, CD8, CD19, and CD56 (eBioscience, San Diego, USA). After a 30-minute incubation in the dark, the cells were washed, centrifuged, and analyzed using a FACSCanto II flow cytometer (BD, Heidelberg, Germany). FlowJo software (Treestar Inc., Ashland, USA) was used for subsequent analysis.

Cell populations were identified based on antibody positivity and doublet exclusion as follows: T cells were defined as CD3^+^CD56^−^, with further analysis for CD4^+^ and CD8^+^ T cell subsets. Natural killer (NK) cells were defined as CD3^−^CD19^−^CD56^+^, and B cells were defined as CD19^+^. The assay was conducted in accordance with the manufacturer’s instructions, with each sample being stained and analyzed following standardized procedures.

### 2.5 Routine blood and cytokine tests

Routine blood tests were performed in the hospital laboratory using an automated hematology analyzer (Sysmex XN-2000, Sysmex Corporation). Peripheral blood samples (2-3 mL) were collected in tubes containing anticoagulant (e.g., EDTA), and processed according to the manufacturer’s instructions. Blood samples were analyzed using the XN-2000 fully automated hematology analyzer kit (model: XN-02, Sysmex Corporation). The test parameters included white blood cell count (WBC), neutrophil count (NEU), lymphocyte count (LYM), and mononuclear cell count (MONO). Absolute cell counts were automatically calculated using the instrument’s differential white blood cell analysis.

Cytokine analysis was conducted using the Cytometric Bead Array (CBA) assay (BD, Heidelberg, Germany) to perform multiplex analysis of plasma cytokines. The cytokines analyzed included Interleukin (IL)-1β, IL-2, IL-6, IL-8, IL-10, and Tumor Necrosis Factor-α (TNF-α). The assay was conducted according to the manufacturer’s instructions.

### 2.6 Statistical analysis

Statistical analyses were conducted using SPSS version 25.0 (IBM, Armonk, USA) and GraphPad Prism software version 9.0 (GraphPad Software, La Jolla, USA). Two-group comparisons were performed using the independent samples *t* tests or chi-square tests. Continuous variables that did not follow a normal distribution were analyzed using the Mann-Whitney U test for two-group comparisons and the Kruskal-Wallis test for multiple-group comparisons. Spearman correlation analysis was employed to assess the correlation between the two variables. Binary logistic regression was utilized to analyze the risk factors influencing prognosis, and Cox regression was applied to examine the independent risk factors affecting the 28-day mortality of patients with sepsis.

Survival curves were generated using the Kaplan-Meier method, with grouping criteria established based on cut-off values determined from receiver operating characteristic (ROC) curve analysis, and differences between groups were assessed using the log-rank test. The area under curve (AUC) was calculated to evaluate the diagnostic efficacy of *miR-146b-5p* and lymphocyte subsets. The indicators used to establish survival curves included the expression levels of *miR-146b-5p* and the absolute counts of Lym, CD3^+^ T cells, CD4^+^ T cells, and CD8+ T cells, as well as CD4^+^ T cell percentage (CD3%), CD4^+^ T cell percentage (CD4%), CD8^+^ T cell percentage (CD8%), B cell percentage (B%), and NK cell percentage (NK%), and the CD4^+^/CD8^+^ ratio. The corresponding cut-off values for these indicators were as follows: *miR-146b-5p* (0.272), Lym (1.24), the absolute counts of CD3^+^ T cells (0.145), CD4^+^ T cells (0.065), CD8^+^ T cells (0.059), CD3% (53.98), CD4% (23.18), CD8% (27.76), B% (5.91), NK% (8.6), and CD4^+^/CD8^+^ ratio (2.54).

## 3 Results

### 3.1 Clinical characteristics of 28-day survivors and nonsurvivors

Patients were divided into two groups based on 28-day mortality. A total of 191 sepsis patients were included in the study, with 117 in the non-survivor group and 74 in the survivor group. The mean age in the non-survivor group was 70.30 ± 13.14 years, with 69.23% of patients being male. In contrast, the survivor group had a mean age of 68.46 ± 17.60 years, with 75.68% of patients being male. No significant differences in age or gender were observed between the survivor and non-survivor groups. Laboratory examinations revealed no significant differences in leukocyte, lymphocyte, neutrophil, and monocyte counts between the two groups. However, nonsurvivor patients exhibited significantly higher APACHE II scores of 24.12 ± 8.14 and SOFA scores of 9.89 ± 4.16 than the survivor group. Additionally, levels of IL-6 and IL-10 were markedly elevated in nonsurvivor patients (IL-6, 1307.91 ± 3254.43 pg/ml; IL-10, 123.94 ± 278.73 pg/ml) compared to survivor patients (IL-6, 379.28 ± 902.33 pg/ml, *p* = 0.018; IL-10, 30.00 ± 73.85 pg/ml, *p* = 0.005) (**Table 1**).

**Table 1:** Clinical characteristics of 28-day survivors and nonsurvivors.

Characteristics	Non-survivors (n=117)	Survivors (n=74)	*P*
Gender (F/M)			0.335
Male	81 (69.23)	56 (75.68)	
Famale	36 (30.77)	18 (24.32)	
Age (y)	70.30 ± 13.14	68.46 ± 17.60	0.521
APACHE II score	24.12 ± 8.14	17.03 ± 7.14	**<0.001**
SOFA score	9.89 ± 4.16	6.62 ± 3.48	**<0.001**
Laboratory parameters			
White blood cell	12.90 ± 9.17	13.12 ± 8.16	0.866
Neutrophil	11.38 ± 8.86	11.06 ± 7.27	0.792
Lymphocyte	0.79 ± 0.63	0.80 ± 1.00	0.984
Monocyte	1.03 ± 3.55	0.61 ± 0.53	0.242
CRP (mg/l)	136.91 ± 69.22	126.22 ± 63.78	0.293
PCT (ng/ml)	24.07 ± 26.17	8.19 ± 9.36	0.102
IL-1β (pg/ml)	10.50 ± 26.83	4.86 ± 6.53	0.078
IL-2 (pg/ml)	2.76 ± 5.00	1.51 ± 1.85	0.115
IL-6 (pg/ml)	1307.91 ± 3254.43	379.28 ± 902.33	**0.018**
IL-8 (pg/ml)	1065.08 ± 2275.30	459.54 ± 978.10	0.219
IL-10 (pg/ml)	123.94 ± 278.73	30.00 ± 73.85	**0.005**
TNF-α (pg/ml)	18.51 ± 48.20	7.69 ± 10.27	0.054

Comparison of demographic, clinical, and laboratory characteristics between 28-day survivors (n = 74) and nonsurvivors (n = 117). Data are presented as median ± standard deviation (SD) or number (percentage) as appropriate. Independent t tests or chi-square tests were used to compare groups. APACHE II score, Acute Physiology and Chronic Health Evaluation II score; SOFA, Sequential Organ Failure Assessment; CRP, C-reactive protein; PCT, procalcitonin. The *p* value indicates the statistical significance of differences between survivors and nonsurvivors, with significant differences highlighted in bold.

### 3.2 Differential expression of *miR-146b-5p* in patients with sepsis and its association with patient survival

**Figure 2:**
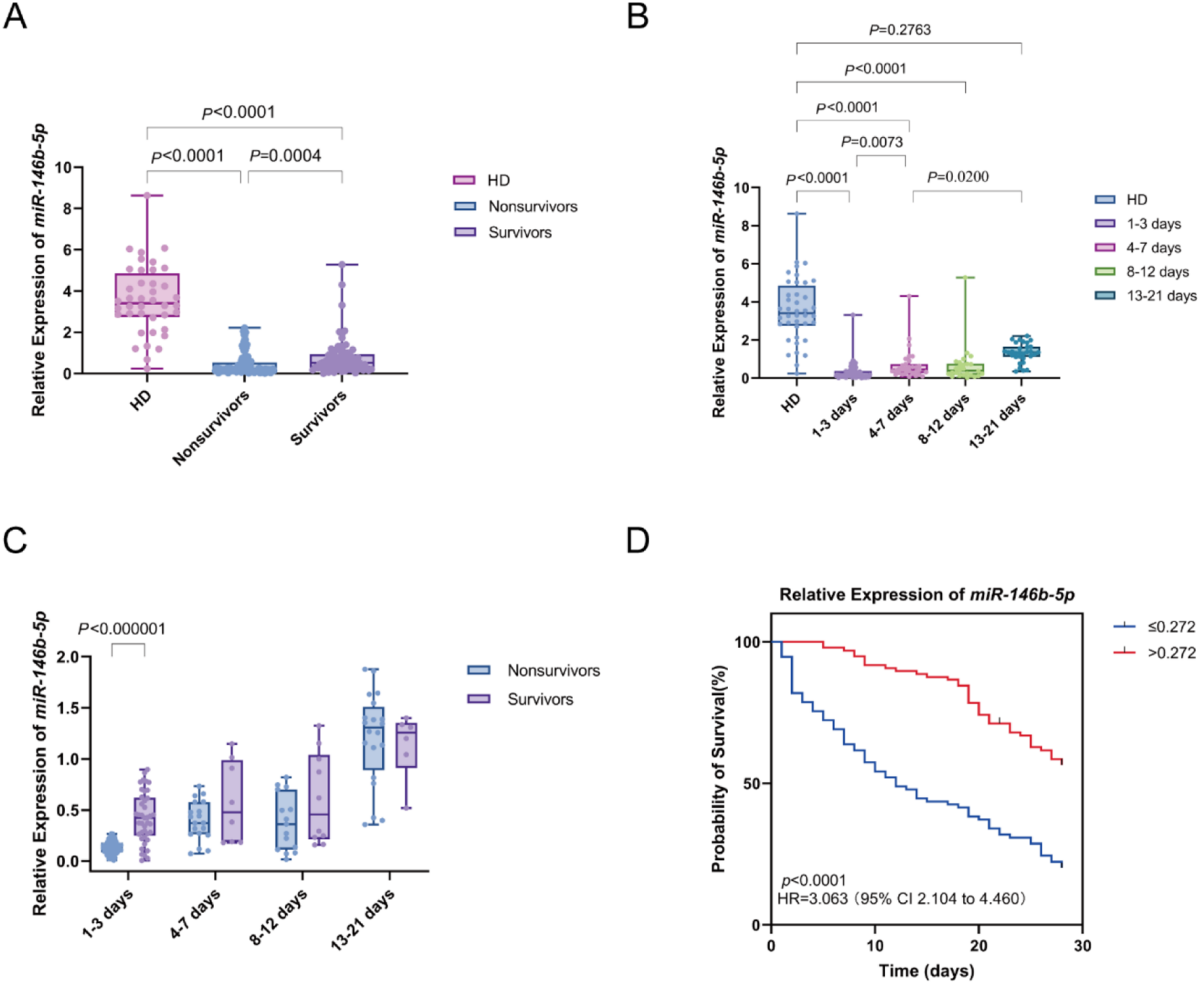
Differential expression of *miR-146b-5p* in sepsis patients and its relationship with survival outcomes. **(A)** Box plots displaying *miR-146b-5p* expression levels in PBMCs from healthy donors (HD) and sepsis patients (nonsurvivors and survivors). **(B)** Trend of *miR-146b-5p* expression levels in PBMCs of sepsis patients at different time intervals(D1-3, D4-7, D8-12, D13-21) Time intervals represent days after admission. **(C)** Comparison of *miR-146b-5p* expression between nonsurvivors and survivors at multiple time points. **(D)** Kaplan-Meier survival curves showing the 28-day survival of sepsis patients classified into high (miR-146b-5p > 0.272) and low (miR-146b-5p ≤ 0.272) expression groups. Each dot represents an individual patient’s expression level. The boxes indicate the lower and upper quartile values with the line indicating the median value, the whiskers indicate the lowest and highest values. The Mann-Whitney U test was used for comparisons between two groups and the Kruskal-Wallis test was applied for analysis of three or more groups. Survival differences were analyzed with log-rank tests. The *p* value indicates the level of statistical significance.

We analyzed the differences in the relative expression of *miR-146b-5p* between sepsis patients and HD. The relative expression of *miR-146b-5p* was significantly lower in the sepsis group than in the HD group. Furthermore, within the sepsis cohort, nonsurvivors exhibited significantly lower *miR-146b-5p* expression compared to survivors (**Figure 2A**).

Peripheral blood samples from sepsis patients were collected at four distinct time intervals: days 1-3 (D1-3), days 4-7 (D4-7), days 8-12 (D8-12), and days 13-21 (D13-21) to assess the dynamic changes in *miR-146b-5p* expression. A comparison of *miR-146b-5p* expression across these time points with baseline levels from the HD group revealed significantly lower expression in the D1-3, D4-7, and D8-12 groups compared to the HD group (all *P* < 0.0001). We observed an increasing trend in the expression of *miR-146b-5p* over time, with levels in the D4-7 group significantly higher than those in the D1-3 group (*p* = 0.0073), the expression in the D13-21 group was greater than that in the D4-7 group (*p* = 0.0200). However, no significant difference was found between the D13-21 group and the HD group (*p* = 0.2763) (**Figure 2B**).

The relative expression of *miR-146b-5p* was compared between the nonsurvivor and survivor groups across the time intervals (D 1-3, D 4-7, D 8-12, and D 13-21). At D1-3, *miR-146b-5p* levels were significantly lower in the nonsurvivor group compared to the survivor group. There is no significant difference in other time intervals. This observation suggests that lower *miR-146b-5p* expression during the early stages of sepsis is associated with an increased risk of mortality **Figure 2C**).

The 28-day prognosis of patients with varying levels of *miR-146b-5p* was evaluated using an optimal cut-off value as the classification criterion. All patients were divided into a high *miR-146b-5p* group (*miR-146b-5p* > 0.272) and a low *miR-146b-5p* group (*miR-146b-5p* ≤ 0.272). Notably, the Kaplan-Meier survival curve indicated that patients with *miR-146b-5p* expression below 0.272 had a significantly higher risk of death compared to those with higher expression (log-rank χ² = 39.46, *p* < 0.001) (**Figure 2D**). Taken together, these findings suggest that lower relative expression of *miR-146b-5p* in PBMCs is associated with increased mortality in septic patients.

### 3.3 Differential expression of lymphocyte subsets in patients with sepsis and its relation to patient survival

**Figure 3:**
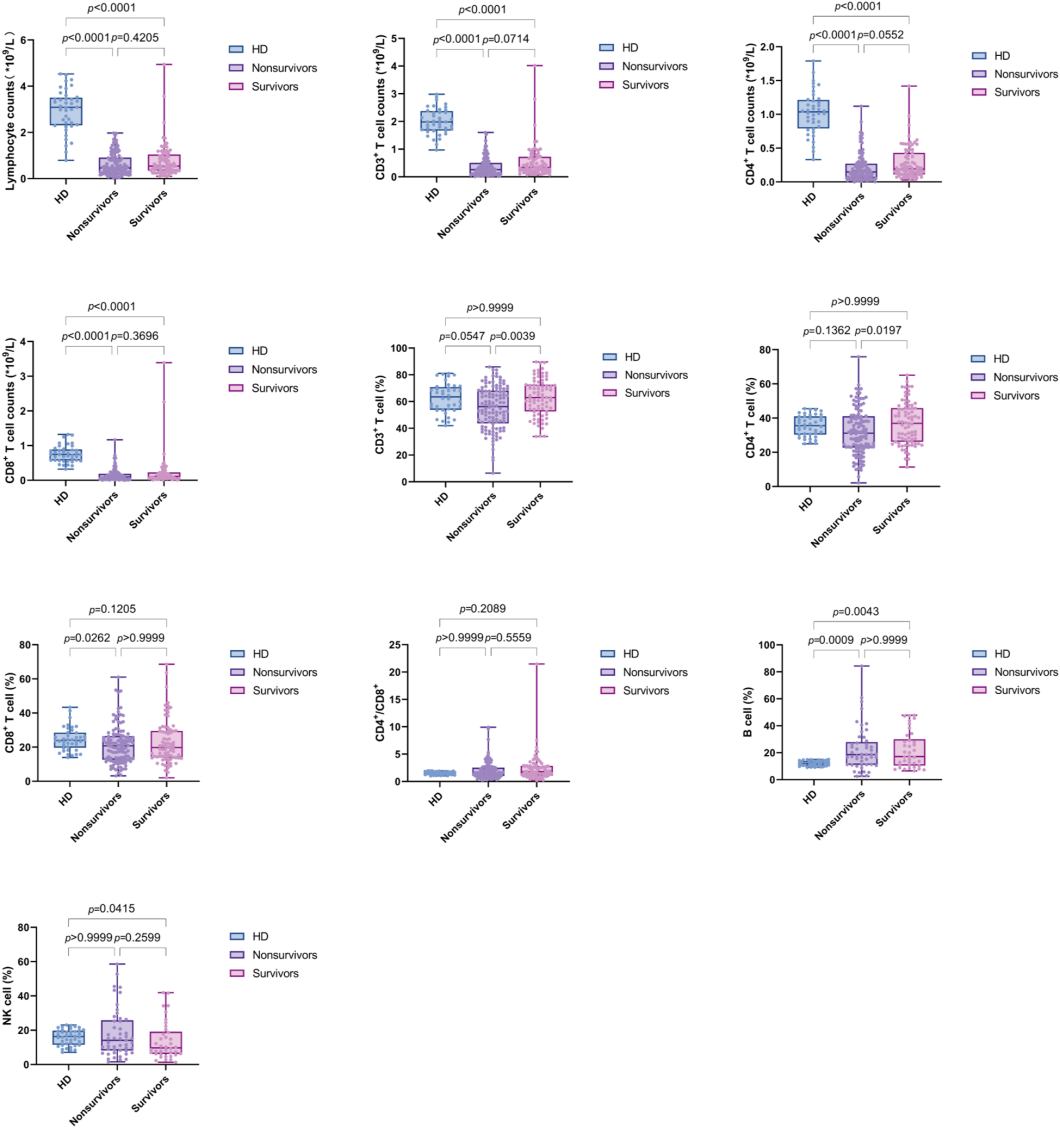
Box plots displaying lymphocyte subsets levels in PBMCs from healthy donors (HD) and sepsis patients (nonsurvivors and survivors). Each dot represents an individual patient’s expression level. The boxes indicate the lower and upper quartile values with the line indicating the median value, the whiskers indicate the lowest and highest values. Group comparisons were performed by Mann-Whitney U test. The *p* value indicates the level of statistical significance.

We compared lymphocyte subsets between sepsis patients and HD group. The absolute counts of lymphocytes (Lym), CD3^+^ T cells, CD4^+^ T cells, and CD8^+^ T cells were significantly lower in sepsis patients compared to HD. However, B% and NK% showed an increasing trend in sepsis patients compared to HD. We further examined the changes in T cell subsets between nonsurvivors and survivors of sepsis patients. The CD3% and CD4% were significantly lower in nonsurvivors compared to survivors. Notably, no significant difference was observed in the CD4^+^/CD8^+^ T cell ratio between sepsis patients and HD, which may be attributed to the concurrent reduction of both CD4^+^ and CD8^+^ T cells in sepsis patients (**Figure 3**).

**Figure 4:**
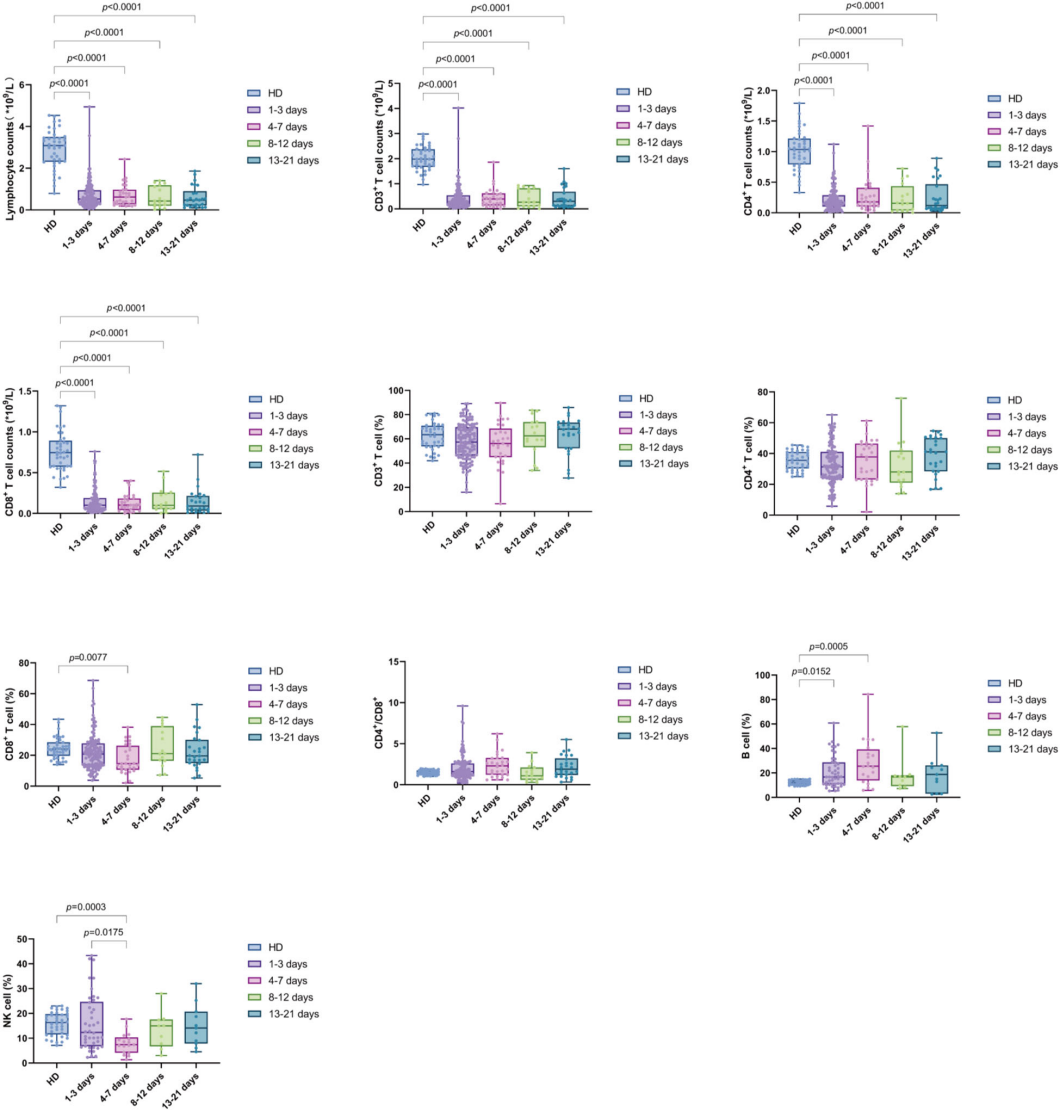
Trend of lymphocyte subsets in PBMCs of sepsis patients at different time intervals (D1-3, D4-7, D8-12, and D13-21). Time intervals represent days after admission. Each dot represents an individual patient’s expression level. The boxes indicate the lower and upper quartile values with the line indicating the median value, the whiskers indicate the lowest and highest values. Differences between groups were calculated using Kruskal-Wallis test. The *p* value indicates the level of statistical significance. indicates the level of statistical significance.

We further compared the changes in the four time intervals. The results indicated that the absolute counts of lymphocytes, CD3^+^ T cells, CD4^+^ T cells, and CD8^+^ T cells were significantly lower than those in the HD group during all periods (*p* value were less than 0.0001). B% was reduced in the D1-3 and D4-7 groups compared to HD (*p* = 0.0152 and *p* = 0.0005, respectively). Additionally, CD8% and NK% were lower in the D4-7 group compared to HD (*p* = 0.0077 and *p* = 0.0003, respectively). No significant differences were observed in the CD4%, CD3%, and CD4^+^/CD8^+^ T cell ratio compared to HD. Furthermore, NK% was significantly lower in the D4-7 group than in the D1-3 group (*p* = 0.0175). Furthermore, dynamic analysis revealed that, compared to HD group, used as the baseline, the total lymphocyte count, as well as the absolute counts of CD3^+^ T cells, CD4^+^ T cells, and CD8^+^ T cells, were significantly lower across all four time points (D1-3, D4-7, D8-12, and D13-21). The percentage of B cells increased significantly during the early stage of sepsis, within the first week, and subsequently declined. In contrast, the percentage of NK cells showed the opposite pattern, with a significant decrease in the first week after admission, followed by a gradual increase over time. No significant changes were observed in the proportions of other lymphocyte subsets, including CD3^+^ T cells, CD4^+^ T cells, and CD8^+^ T cells, across the different time intervals (**Figure 4**).

**Figure 5:**
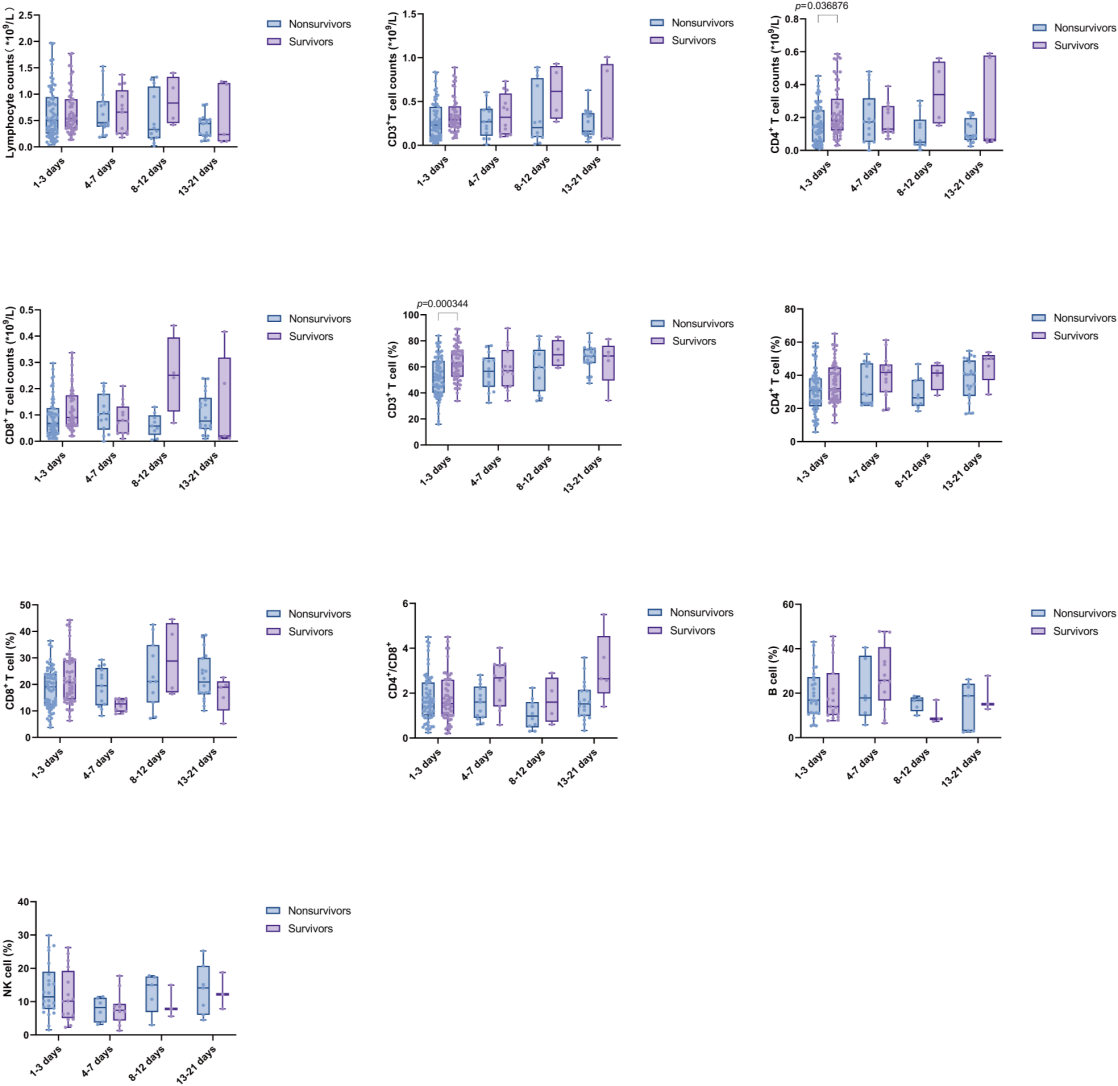
Comparison of Lymphocyte subsets between nonsurvivors and survivors at multiple time points. Time intervals represent days after admission. Each dot represents an individual patient’s expression level. The boxes indicate the lower and upper quartile values with the line indicating the median value, the whiskers indicate the lowest and highest values. Differences between groups were calculated using Mann-Whitney U test. The *p* value indicates the level of statistical significance.

The expression of lymphocyte subsets in both the nonsurvivor and survivor groups was compared across the intervals D1-3, D4-7, D8-12, and D13-21. During the D1-3 period, the absolute counts of CD4^+^ T cells and CD3% were significantly higher in the survivor group compared to the nonsurvivor group. However, the differences in absolute counts of Lym, CD4^+^ T cells, CD8^+^ T cells, and CD3^+^ T cells and CD4%, CD8%, B cell%, NK%, and CD4^+^/CD8^+^ T cell ratio were not statistically significant. This finding suggests that in the early stage (within one week of admission), the levels of lymphocyte subsets in the two groups were similar; while in the latter two time periods, the survival group had a higher trend than the nonsurvivor group, although this difference was not statistically significant (**Figure 5**).

**Figure 6:**
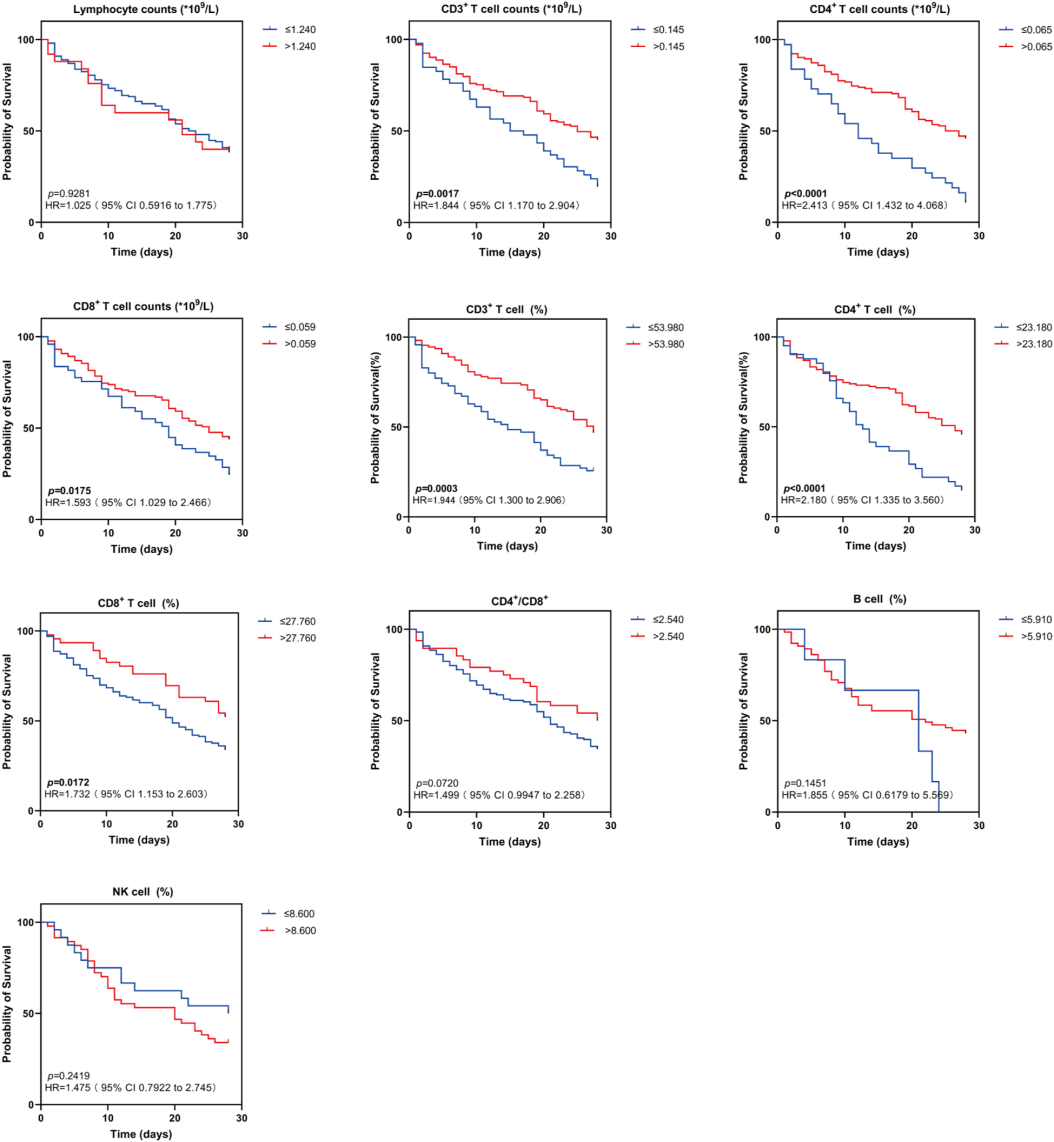
Kaplan-Meier survival curves for two groups of patients with different lymphocyte subsets levels. Kaplan-Meier survival curves demonstrating lymphocyte subsets (Lym, CD3+ T cell counts, CD4+ T cell counts, CD8+ T cell counts, CD3+ T cell (%), CD4+ T cell (%), CD8+ T cell (%), CD4+/CD8+, B cell (%), NK cell (%) in sepsis patients in the high and low levels groups for 28-day survival. HR indicates hazard ratio; 95% CI, 95% confidence interval. Survival differences were analyzed with log-rank tests. The *p* value indicates the level of statistical significance.

Kaplan-Meier (K-M) curves showed that among lymphocyte subsets, patients in the group with high expression of CD3%, CD4%, and CD8%, as well as in the group with high expression of the absolute counts of CD3^+^ T cells, CD4^+^ T cells, and CD8^+^ T cells had a significantly lower mortality rate than patients in the group with low expression of these lymphocytes after 28 days (χ2 = 13.16, *p* = 0.0003; χ2 = 16.09, *p* < 0.0001; χ2 = 5.676, *p* = 0.0172; χ2 = 9.894, *p* = 0.0017; χ2 = 20.06, *p* < 0.0001; χ2 = 5.642, *p* = 0.0175). No significant differences were observed between the two groups regarding B%, NK%, CD4^+^/CD8^+^ T cell ratio, and Lym (**Figure 6**).

### 3.4 Correlation study of relative expression of *miR-146b-5p* and lymphocyte subsets and disease severity in patients with sepsis

Spearman correlation coefficients were employed to examine the relationship between *miR-146b-5p* expression levels, lymphocyte subsets, and APACHE II and SOFA scores to assess their correlation with disease severity. Notably, CD3% and CD4% exhibited a negative correlation with the APACHE II scores in sepsis patients (r = -0.214, *p* = 0.0103; r = -0.237, *p* = 0.0043), while CD3% showed a negative correlation with the SOFA score (r = -0.242, *p* = 0.0287). When analyzing the relative expression of *miR-146b-5p*, no significant correlation was observed with either the APACHE II or SOFA scores in the overall assessment. During days 1 to 3 (D1-3), the relative expression of *miR-146b-5p*, CD3%, and CD4% demonstrated negative correlations with the APACHE II scores in sepsis patients (r = -0.224, *p* = 0.0241; r = -0.280, *p* = 0.0046; r = -0.240, *p* = 0.0156). From days 4 to 7 (D4-7), a negative correlation was identified between *miR-146b-5p* expression and the CD4^+^/CD8^+^ T cell ratio with the APACHE II scores (r = -0.435, *p* = 0.0338; r = -0.592, *p* = 0.0426), while positive correlations were observed between CD8% and NK% with the SOFA scores (r = 0.677, *p* = 0.0155; r = 0.681, *p* = 0.0211). During days 8 to 12 (D8-12), no associations were found between *miR-146b-5p* expression levels and lymphocyte subsets with the APACHE II and SOFA scores. From days 13 to 21 (D13-21), absolute counts of CD4^+^ T cells were positively correlated with the SOFA score (r = 0.900, *p* = 0.0374; r = 1.000, *p* < 0.0001) and negatively correlated with NK% (r = -1.000, *p* < 0.0001). These findings suggest that lower expression levels of *miR-146b-5p*, CD3%, and CD4% are associated with higher APACHE II scores, which indicate a more severe condition in sepsis patients. However, it is important to note that the strength of these correlations is relatively weak (**Table 2**).

**Table 2:** Correlation between *miR-146b-5p* expression and lymphocyte subsets in relation to disease severity.

Variables	Time intervals	*miR-146b-5p*		lymphocyte counts		CD3^+^ T cell counts		CD4^+^ T cell counts		CD8^+^ T cell counts		CD3^+^ T cell (%)		CD4^+^ T cell (%)		CD8^+^ T cell (%)		CD4^+^/CD8^+^		B cell (%)		NK cell (%)	
		r	*p*	r	*p*	r	*p*	r	*p*	r	*p*	r	*p*	r	*p*	r	*p*	r	*p*	r	*p*	r	*p*
**APACHE II score**	**total**	-0.152	0.0585	0.00951	0.9103	-0.0829	0.3252	-0.114	0.1763	-0.0335	0.6911	-0.214	**0.0103**	-0.237	**0.0043**	-0.0603	0.4747	-0.103	0.2209	0.0886	0.4757	0.201	0.1031
	**1-3 days**	-0.224	**0.0241**	0.0385	0.7025	-0.0324	0.7476	-0.0801	0.4259	-0.00694	0.9451	-0.280	**0.0046**	-0.240	**0.0156**	-0.112	0.2650	-0.0497	0.6213	0.201	0.1964	0.218	0.1606
	**4-7 days**	-0.435	**0.0338**	-0.136	0.6150	-0.256	0.3380	-0.349	0.1847	-0.0882	0.7453	-0.344	0.1924	-0.386	0.1393	0.135	0.6189	-0.478	0.0610	0.00705	0.9826	-0.332	0.2924
	**8-12 days**	-0.146	0.4947	-0.154	0.6325	-0.144	0.6561	-0.0525	0.8712	-0.263	0.4094	-0.0420	0.8968	0.0560	0.8627	-0.382	0.2207	0.196	0.5405	-0.0286	0.9572	0.143	0.7872
	**13-21 days**	-0.0138	0.9500	0.0616	0.8343	0.0792	0.7878	0.165	0.5729	-0.0352	0.9049	0.0858	0.7705	0.209	0.4733	-0.00440	0.9881	0.0573	0.8458	0.143	0.7872	-0.314	0.5441
**SOFA score**	**total**	-0.151	0.1458	0.102	0.3620	0.00322	0.9771	-0.0171	0.8791	0.0592	0.5974	-0.242	**0.0287**	-0.163	0.1428	-0.0670	0.5500	-0.0827	0.4602	0.112	0.4322	0.171	0.2296
	**1-3days**	-0.234	0.0669	0.170	0.1876	0.0558	0.6668	0.0421	0.7452	0.0429	0.7407	-0.318	0.0119	-0.131	0.3088	-0.240	0.0599	0.0599	0.6436	0.221	0.2159	0.0511	0.7775
	**4-7days**	-0.330	0.1551	-0.207	0.5190	-0.182	0.5709	-0.300	0.3442	0.156	0.6291	-0.0268	0.9341	-0.424	0.1692	0.677	**0.0155**	-0.592	**0.0426**	-0.462	0.1529	0.681	**0.0211**
	**8-12days**	0.305	0.2344	-0.103	0.8696	-0.0513	0.9347	-0.0513	0.9347	0.0513	0.9347	0.564	0.3217	0.308	0.6144	-0.154	0.8048	0.308	0.6144	-0.205	0.7406	0.103	0.8696
	**13-21days**	0.0421	0.8966	0.900	**0.0374**	0.700	0.1881	1.000	**<0.0001**	0.400	0.5046	0.000	1.0000	0.300	0.6238	0.300	0.6238	-0.100	0.8729	0.400	0.6000	-1.000	**<0.0001**

Spearman correlation analysis was used to evaluate the correlation between *miR-146b-5p* expression and lymphocyte subsets, and disease severity scoring systems (APACHE II and SOFA scores). APACHE II score, Acute Physiology and Chronic Health Evaluation II score; SOFA score, Sequential Organ Failure Assessment score. The symbol ‘r’ denotes the correlation coefficient, while the *p* value indicates the level of statistical significance, with significant differences are highlighted in bold.

### 3.5 Logistic regression analysis of *miR-146b-5p* and lymphocyte subsets as independent predictors of sepsis

Univariate logistic regression results indicated that the expression level of *miR-146b-5p*, CD3%, CD4%, APACHE II scores, and SOFA scores were independent risk factors for sepsis prognosis (*p* = 0.003, 0.002, 0.037, 0.001, 0.001). Additionally, multivariate logistic regression results demonstrated that the expression level of *miR-146b-5p* and CD3% were significant risk factors influencing the prognosis of sepsis patients. The above findings suggest that *miR-14bb-5p* expression and CD3% may serve as reliable biomarkers for predicting disease outcomes (*p* = 0.027, 0.032) (**Table 3**).

**Table 3:** Logistic regression analysis (univariate and multivariate) of *miR-146b-5p* and lymphocyte subsets as independent predictors of sepsis.

Variables	Univariate analysis					Multivariate analysis				
	β	S.E	Z	*P*	OR (95%CI)	β	S.E	Z	*P*	OR (95%CI)
*miR-146b-5p*	-0.78	0.27	-2.94	**0.003**	0.46 (0.27 ~ 0.77)	-0.54	0.25	-2.22	**0.027**	0.58 (0.36 ~ 0.94)
APACHE II score	0.13	0.03	4.66	**0.001**						
SOFA score	0.22	0.06	3.59	**0.001**						
B cell (%)	0.00	0.02	0.10	0.923	1.00 (0.97 ~ 1.03)					
NK cell (%)	0.02	0.02	1.15	0.249	1.02 (0.98 ~ 1.06)					
CD3^+^ T cell (%)	-0.03	0.01	-3.06	**0.002**	0.97 (0.95 ~ 0.99)	-0.03	0.01	-2.15	**0.032**	0.97 (0.95 ~ 0.99)
CD4^+^ T cell (%)	-0.02	0.01	-2.09	**0.037**	0.98 (0.95 ~ 0.99)	-0.01	0.01	-0.34	0.732	0.99 (0.97 ~ 1.02)
CD8^+^ T cell (%)	-0.01	0.01	-1.01	0.313	0.99 (0.96 ~ 1.01)					
CD3^+^ T cell counts(*109/L)	-0.75	0.39	-1.94	0.053	0.47 (0.22 ~ 1.01)					
CD4^+^ T cell counts(*109/L)	-1.18	0.73	-1.61	0.106	0.31 (0.07 ~ 1.29)					
CD8^+^ T cell counts(*109/L)	-0.88	0.61	-1.44	0.149	0.41 (0.12 ~ 1.37)					
Lymphocyte counts(*109/L)	-0.39	0.26	-1.46	0.145	0.68 (0.41 ~ 1.14)					
CD4^+^/CD8^+^	-0.09	0.08	-1.15	0.251	0.92 (0.79 ~ 1.06)					

Logistic regression analysis (univariate and multivariate) was used to analysis the miR-146b-5p and lymphocyte subsets as independent predictors of sepsis.

The p value indicates the level of statistical significance, with signifcant diferences highlighted in bold.

### 3.6 Cox proportional hazard model of relative expression of *miR-146b-5p* and 28-day mortality

The Cox proportional hazard model with time-dependent covariates indicated that, in the univariate analysis, the relative expression of *miR-146b-5p* in PBMCs, APACHE II scores, SOFA scores, CD3%, CD4%, procalcitonin (PCT) and IL-6 were significantly associated with the 28-day mortality. The hazard ratios (HR) for these variables were as follows: relative expression of *miR-146b-5p* (HR = 0.452, 95% CI 0.297-0.690), APACHE II scores (HR =1.064, 95% CI 1.041-1.087), SOFA scores (HR = 1.155, 95% CI 1.077-1.239), CD3% ((HR = 0.976, 95% CI 0.964-0.988), CD4% with hazard ratios of (HR =0.981, 95% CI 0.966-0.996), PCT (HR = 1.003, 95% CI 1.001-1.006), and IL-6 (HR = 1.000, 95% CI 1.000-1.000) were significantly associated with 28-day mortality in the univariate analysis. In the multivariate analysis, the relative expression of *miR-146b-5p* and the APACHE II scores remained significant, with hazard ratios of 0.145 (95% CI, 0.044-0.476) and 1.103 (95% CI, 1.027-1.184). These findings suggest that *miR-146b-5p* expression and APACHE II scores may serve as more reliable predictors of disease outcomes (**Table 4**).

**Table 4:** Cox regression analysis (univariate and multivariate) of factors associated with 28-day mortality in patients with sepsis.

Variables	Univariate analysis			Multivariate analysis		
	HR	95% CI	*P*	HR	95% CI	*P*
*miR-146b-5p*	0.452	0.297-0.690	**<0.001**	0.145	0.044-0.476	**0.001**
APACHE II score	1.064	1.041-1.087	**<0.001**	1.103	1.027-1.184	**0.007**
SOFA score	1.155	1.077-1.239	**<0.001**	1.055	0.933-1.193	0.391
CD3^+^ T cell (%)	0.976	0.964-0.988	**<0.001**	0.944	0.894-0.008	0.041
CD4^+^ T cell (%)	0.981	0.966-0.996	**0.013**	1.049	1.001-1.099	0.044
CD3^+^ T cell counts	0.578	0.332-1.006	0.053			
CD4^+^ T cell counts	0.403	0.147-1.108	0.078			
PCT	1.003	1.001-1.006	**0.004**	1.000	0.993-1.007	0.934
IL-6	1.000	1.000-1.000	**0.000**	1.000	0.100-1.000	0.430

Cox regression analysis (univariate and multivariate) was used to analyze the factors associated with 28-day mortality in patients with sepsis. HR indicates the hazard ratio, and 95% CI denotes the 95% confidence interval. APACHE II score, Acute Physiology and Chronic Health Evaluation II score; SOFA score, Sequential Organ Failure Assessment score; CRP, C-reactive protein; PCT, procalcitonin. The *p* value indicates the level of statistical significance, with significant differences highlighted in bold.

### 3.7 Correlation analysis of relative expression of *miR-146b-5p* with lymphocyte subsets

We used Spearman correlation analysis to examine the relationship between *miR-146b-5p* and lymphocyte subsets. Overall, *miR-146b-5p* exhibited a positive correlation with CD3% and CD4% (r = 0.242, *p* = 0.001; r = 0.155, *p* = 0.038). During the D1-3 period, *miR-146b-5p* showed positive correlations with CD3%, CD4%, and absolute counts of CD3^+^ T and CD4^+^ T cells (r = 0.312, *p* = 0.001; r = 0.204, *p* = 0.039; r = 0.233, *p* = 0.028; r = 0.233, *p* = 0.018). In the D4-7 period, *miR-146b-5p* was positively correlated with CD3% and CD4% (r = 0.453, *p* = 0.045; r = 0.522, *p* = 0.018). Conversely, during D8-12, *miR-146b-5p* exhibited negative correlations with CD4%, NK%, and CD4^+^/CD8^+^ T cell ratios (r = -0.566, *p* = 0.001; r = -0.658, *p* = 0.014; r = -0.658, *p* = 0.014). Moreover, at D13-21, *miR-146b-5p* was not correlated with any lymphocyte subsets. These findings indicate that *miR-146b-5p* is correlated with certain lymphocyte subsets, although the correlation is relatively weak, suggesting that changes in *miR-146b-5p* expression may affect the immune status of patients (**Table 5**).

**Table 5:** Correlation study of *miR-146b-5p* expression with lymphocyte subsets.

Variable	Total		1-3days		4-7days		8-12days		13-21days	
	r	*p*	r	*p*	r	*p*	r	*p*	r	*p*
CD3^+^ T cell (%)	0.242	**0.001**	0.312	**0.001**	0.453	**0.045**	0.209	0.494	0.185	0.411
CD4^+^ T cell (%)	0.155	**0.038**	0.204	**0.039**	0.522	**0.018**	-0.566	**0.044**	-0.232	0.299
CD8^+^ T cell (%)	0.092	0.219	0.110	0.269	0.150	0.527	0.505	0.078	0.333	0.130
CD3^+^ T cell counts	-0.053	0.484	0.233	**0.028**	-0.121	0.611	-0.093	0.762	-0.094	0.676
CD4^+^ T cell counts	-0.061	0.414	0.233	**0.018**	-0.144	0.538	-0.082	0.789	-0.238	0.287
CD8^+^ T cell counts	0.043	0.572	0.150	0.130	-0.068	0.777	0.247	0.415	0.038	0.867
B cell (%)	0.106	0.380	0.008	0.970	-0.191	0.574	0.257	0.623	**0.000**	1.000
NK cell (%)	-0.051	0.501	0.094	0.344	-0.236	0.316	-0.658	**0.014**	-0.380	0.081
lymphocyte counts	-0.020	0.793	0.038	0.702	-0.138	0.637	0.115	0.751	-0.260	0.314
CD4^+^/CD8^+^	-0.051	0.501	0.094	0.344	-0.236	0.316	-0.658	**0.014**	-0.380	0.081

Spearman correlation analysis was used to evaluate the correlation between *miR-146b-5p* expression and lymphocyte subsets. The symbol ‘r’ denotes the correlation coefficient, while the *p* value indicates the statistical significance of the differences between correlations for each group, with significant differences are highlighted in bold.

### 3.8 ROC curves for variables predicting 28-day mortality risk in sepsis

**Figure 7:**
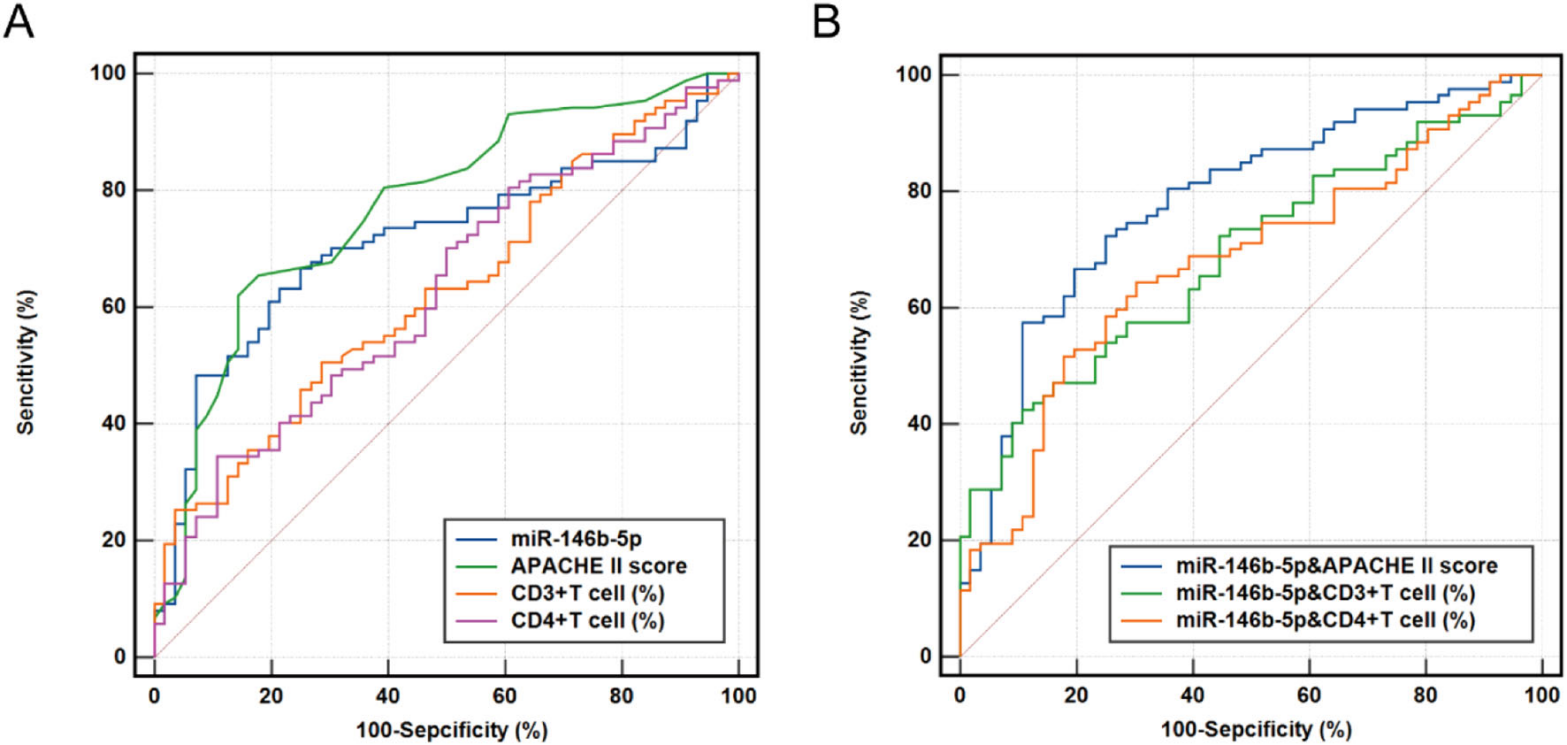
Receiver operating characteristic (ROC) curve for predicting 28-day mortality in patients with sepsis. **(A)** ROC curve of *miR-146b-5p* expression, APACHE II scores, CD3%, and CD4% levels. **(B)** ROC curve of *miR-146b-5p* expression, in combined with the APACHE II scores, CD3%, and CD4%. ROC curves were plotted using the true positive rate (sensitivity) against the false positive rate (1- specificity) at various cut-off points for miR-146b-5p expression, APACHE II scores, CD3%, and CD4% in patients with sepsis. The areas under the ROC curves were calculated to show the values of AUC. The *p* value indicates the level of statistical significance.

The logistic regression analysis and Cox regression analysis were used to evaluate the predictive value of serum *miR-146b-5p*, APACHE II scores, CD3%, and CD4% levels for the prognosis of patients with sepsis (**Figure 7A**). The AUC values for *miR-146b-5p*, APACHE II scores, CD3%, and CD4% in predicting the prognosis of sepsis patients were 0.691, 0.761, 0.633, and 0.611, respectively (**Table 6**). To further improve predictive accuracy, logistic regression analysis was used to calculate the combined prediction probability of *miR-146b-5p*, APACHE II scores, CD3%, and CD4%. ROC curve analysis was then conducted to evaluate the prognostic value of these combined indicators in sepsis patients (**Figure 7B**). The AUC results for the three indicators were 0.785, 0.765, and 0.646, respectively, all of which were superior to those predicted by any single index (**Table 6**). Notably, the combination of *miR-146b-5p* and the APACHE II scores yielded the highest AUC.

**Table 6:** Areas under the ROC curves for predicting 28-day mortality in septic patients.

Parameter	AUC	Sensitivity (%)	Specificity (%)	95% CI	*P*	Cut off
*miR-146b-5p*	0.691	62.4	74.3	0.62 - 0.76	<0.001	0.272
APACHE II score	0.761	64.9	82.0	0.69 - 0.83	<0.001	20
CD3^+^ T cell (%)	0.633	47.4	74.7	0.56 - 0.70	0.001	53.98
CD4^+^ T cell (%)	0.611	31.0	91.1	0.54 - 0.68	0.006	23.18
*miR-146b-5p*& APACHE II score	**0.785**	81.9	65.6	0.71 - 0.85	<0.001	0.531
*miR-146b-5p*& CD3^+^ T cell (%)	0.765	40.0	88.4	0.60 - 0.74	<0.001	0.713
*miR-146b-5p*& CD4^+^ T cell (%)	0.646	49.1	81.2	0.57 - 0.72	<0.001	0.680

APACHE II score, Acute Physiology and Chronic Health Evaluation II score; AUC, area under the curve; CI, confidence interval; Cut off: optimal threshold. The *p* value indicates the level of statistical significance, with significant differences highlighted in bold.

## 4 Discussion

The pathogenesis of sepsis involves complex systemic inflammatory network effects, genetic polymorphisms, immune dysfunction, coagulation abnormalities, tissue damage, and abnormal host responses to different infectious pathogenic microorganisms and their toxins, which are closely related to multi-system and multi-organ pathophysiological alterations in the organism (30, 31). As a complex systemic inflammatory response syndrome triggered by infection, its pathological process undergoes a dynamic change from hyperinflammatory response to immunosuppression, the latter being one of the key factors leading to high morbidity and mortality (32–37).

Lymphocyte subsets reflect the immunity of the organism, T-lymphocytes, play a central role in mediating cellular immunity and are key indicators of immune status in sepsis (13). CD3^+^ T cells represent the total number of mature T-lymphocytes, are essential for adaptive immune responses. Double-negative T cells (DNs, CD^-^CD8^-^), which are predominantly localized in the skin and mucosal tissues, participating in innate immunity and forming the first line of defense against infections. CD4^+^ T cells (helper T cells) are responsible for relaying pathogenic signals, while CD8^+^ T cells (cytotoxic T cells) directly eliminate infected or abnormal cells through cytotoxic mechanisms (38). A reduction in CD3^+^ T cells, CD4^+^ T cells, and CD8^+^ T cells indicate suppressed immune function, and abnormalities in immune function (e.g., lymphopenia) alongside an imbalance of inflammatory factors have been suggested to play a significant role in the development of sepsis. However, it is important to note that the predictive value of individual lymphocyte subsets for clinical outcomes remains limited. A comprehensive evaluation of immune markers, including lymphocyte subsets, cytokine levels, and other inflammatory mediators, is necessary to accurately assess immune status and predict prognosis in sepsis patients.

In recent years, miRNAs have emerged as critical regulators of inflammation and immune responses, making them a promising area of research in sepsis (20, 39–41). Interestingly, the same miRNA can exhibit different functions in different diseases. Studies have found that *miR-146b-5p* is associated with a variety of diseases, including sepsis, cervical cancer, and hepatocellular carcinoma (HCC) (42–44). In HCC, *miRNAs* such as *miR-15a, miR-125b, and miR-122* have been proposed as potential biomarkers and therapeutic targets (45, 46). Recent studies have highlighted the diagnostic and prognostic potential of miRNA signatures in sepsis. Yao et al. reported that circulating levels of *miR-25* were significantly lower in sepsis patients compared to those with systemic inflammatory response syndrome (SIRS), and lower *miR-25* levels were associated with worse clinical outcomes (47). Formosa A et al. found that *miR-182*, *miR-143*, *miR-145*, *miR-146a*, *miR-150*, and *miR-155* were dysregulated in sepsis patients, and dysregulated *miRNAs* have immunological associations with clinical disease in sepsis, especially, the decrease in *miR-146a* correlated with elevated IL-6 levels and monocyte proliferation, indicating immunological associations with clinical disease in sepsis (20, 39, 48–50). Martin Jouza et al. identified several miRNAs independently validated by different teams in particular, which are *miR-16a*, *miR-16*, *miR-96-5p*, *miR-141*, *miR-181a*, and *miR-1184* as having strong potential for early diagnosis of neonatal sepsis (51). This study focuses on *miR-146b-5p*, and through time course analysis, it reveals its dynamic changes during the early and subsequent stages of sepsis. It was found that *miR-146b-5p* remains at a consistently lower level in nonsurvivors and shows a certain correlation with the dynamic changes of lymphocyte subsets, providing a new perspective for understanding immune dysregulation in sepsis.

In this study, we analyzed ICU patients with sepsis and found no significant differences in age, gender, or baseline laboratory parameters (e.g., leukocyte, lymphocyte, neutrophil, and monocyte counts) between survivors and nonsurvivors. However, nonsurvivors exhibited higher APACHE II and SOFA scores, along with elevated levels of IL-6 and IL-10, highlighting the utility of these scores as indicators of disease severity and prognosis in critically ill patients (52–55).

We further investigated the differential expression and dynamic trends of *miR-146b-5p* and lymphocyte subset levels in patients with sepsis, comparing nonsurvivors to survivors. Our results revealed that *miR-146b-5p* levels were significantly lower in sepsis patients compared to healthy controls during the early stages of the disease, with nonsurvivors exhibiting even lower levels than survivors. Notably, *miR-146b-5p* levels began to recover after two weeks, suggesting a potential role in the immune response to sepsis. We also observed a weak negative correlation between *miR-146b-5p* levels and APACHE II scores during the early stages of sepsis, further supporting its potential as a prognostic marker. Survival analysis indicated that patients with *miR-146b-5p* levels below 0.272 had a significantly higher mortality rate (HR = 3.063), underscoring the clinical relevance of this miRNA in sepsis prognosis.

Our findings also revealed significant abnormalities in immune function among sepsis patients, particularly in the quantity and distribution of lymphocyte subsets. Compared to HD, sepsis patients exhibited markedly reduced absolute counts of Lym, CD3^+^ T cells, CD4^+^ T cells, and CD8^+^ T cells, as well as decreased B% and NK%. These findings are consistent with previous studies demonstrating lymphopenia and immune suppression in sepsis patients. Longitudinal analysis further revealed that CD3^+^, CD4^+^, and CD8^+^ T cell counts remained lower in sepsis patients than in healthy controls at all time points during the early phase of the disease. Additionally, differences in CD4% and CD3%between survivors and nonsurvivors may reflect the severity of immune dysfunction and its impact on clinical outcomes. Although the correlations between CD3%, CD4%, and APACHE II scores were weak, they suggest that immune cell percentages may serve as indirect markers of disease severity in sepsis patients.

Importantly, we found that peripheral blood levels of *miR-146b-5p* were significantly lower in nonsurviving sepsis patients compared to HD, and these levels were positively correlated with CD3% and CD4%. This suggests a potential link between *miR-146b-5p* expression and immune function in sepsis. Patients with reduced lymphocyte subsets also exhibited lower *miR-146b-5p* levels compared to those with normal lymphocyte counts, further supporting the role of *miR-146b-5p* in immune regulation. These findings highlight the potential of *miR-146b-5p* as a novel therapeutic target for sepsis. The precise mechanism by which *miR-146b-5p* regulates T cell function in sepsis has yet to be fully elucidated. Our research group has currently validated *in vitro* that *miR-146b-5p* can directly and positively regulate the expression of the target gene NF-κB in macrophages co-induced by LPS and ATP. This regulation occurs through the promotion of pyroptosis-related proteins such as GSDMD, caspase-11, and ASC, which further enhances the release of inflammatory factors IL-6 and IL-1β, thereby participating in cellular pyroptosis. These findings indicate that miRNA can regulate cellular pyroptosis in sepsis by targeting proteins involved in pyroptosis-related signaling pathways (56). In our study, we observed a lower relative expression of *miR-146b-5p* and higher levels of IL-6 in non-survivors, suggesting that *miR-146b-5p* may influence sepsis prognosis by modulating inflammatory responses. The NF-κB pathway plays a central role in T cell activation and the production of inflammatory factors, indicating that *miR-146b-5p* may regulate T cell function through the potential NF-κB/IL-6 pathway. Future studies are needed to validate these hypotheses and identify specific targets of *miR-146b-5p* in the regulation of T cell function in sepsis, which would provide new therapeutic targets for improving immune function and clinical outcomes in sepsis patients.

To identify independent risk factors influencing sepsis prognosis, we employed binary logistic regression (including univariate and multivariate analyses) and Cox regression (also incorporating univariate and multivariate analyses). These analyses provide a comprehensive evaluation of clinical prognostic factors and offer insights for personalized treatment strategies. Finally, we utilized ROC curves to assess the predictive value of *miR-146b-5p*, lymphocyte subsets, and APACHE II scores in sepsis prognosis. Our results demonstrated that *miR-146b-5p* levels effectively predicted patient prognosis, exhibiting the largest AUC in the ROC analysis. The diagnostic efficacy was further enhanced when *miR-146b-5p* was combined with the APACHE II scores, suggesting its potential utility in clinical practice. However, the predictive value of lymphocyte subsets requires further validation.

The limitations of our research should be noted. Firstly, our research was conducted at a single center in Chongqing, China, which may limit the applicability of our findings to other regions of China or different countries. To mitigate this limitation, we strictly adhered to the inclusion and exclusion criteria, ensuring a certain degree of universality in our data. Additionally, as the Second Affiliated Hospital of Chongqing Medical University is a teaching hospital, the cases we collected can, to some extent, reflect the situation of sepsis patients in other affiliated hospitals of Chongqing Medical University. Secondly, due to resource constraints, the sample size we collected was limited. Therefore, it is necessary to conduct large-scale, multi-center, randomized controlled trials to further validate our findings and comprehensively assess their universality and specificity in diverse populations.

## 5 Conclusion

In conclusion, our study highlights the significant downregulation of *miR-146b-5p* during the early stages of sepsis and its potential as a biomarker for assessing disease severity and prognostic risk. The combination of *miR-146b-5p* with APACHE II scores improved diagnostic accuracy, underscoring its clinical relevance. Additionally, dynamic monitoring of lymphocyte subsets may aid in evaluating immune status and guiding personalized treatment strategies. The positive correlation between *miR-146b-5p* levels and certain lymphocyte subsets suggests that *miR-146b-5p* may modulate immune responses by influencing T-cell function. However, the precise mechanisms underlying this relationship remain unclear and warrant further investigation through cellular experiments and animal models.

## Data Availability

The original contributions presented in the study are included in the article/supplementary material. Further inquiries can be directed to the corresponding authors.

## Glossary

APACHE: Acute physiology and chronic health evaluation
AUC: Area under the curve
CI: Confidence interval
CRP: C-reactive protein
EDTA: Ethylenediaminetetraacetic acid
HCC: Hepatocellular carcinoma
HD: Healthy donors
HMGB1: High mobility group box 1 protein
IBD: Inflammatory bowel disease
ICU: Intensive care unit
IL-1β: Interleukin-1β
IL-2: Interleukin-2
IL-6: Interleukin-6
IL-8: Interleukin-8
IL-10: Interleukin-10
K-M: Kaplan-meier
LYM: Lymphocyte
MONO: Mononuclear
mRNA: Messenger RNA
NEU: Neutrophil
NK: Natural killer
PBMCs: Peripheral blood mononuclear cells
PCT: Procalcitonin
ROC: Receiver operating characteristic
SIRS: Systemic inflammatory response syndrome
SLE: Systemic lupus erythematosus
SOFA: Sequential organ failure assessment
TNF-α: Tumor necrosis factor alpha
WBC: White blood cell count
